# The Mechanisms of Catalysis by Metallo *β*-Lactamases

**DOI:** 10.1155/2008/576297

**Published:** 2008-06-04

**Authors:** Michael I. Page, Adriana Badarau

**Affiliations:** Department of Chemical and Biological Sciences, University of Huddersfield, Huddersfield HD1 3DH, UK

## Abstract

Class B *β*-lactamases or metallo-*β*-lactamases (MBLs) require zinc ions to catalyse the hydrolysis of *β*-lactam antibiotics such as penicillins, cephalosporins, carbapenems, and cephamycins. There are no clinically useful inhibitors against MBLs which are responsible for the resistance of some bacteria to antibiotics. There are two metal-ion binding sites that have different zinc ligands but the exact roles of the metal-ion remain controversial, and distinguishing between their relative importance is complex. The metal-ion can act as a Lewis acid by co-ordination to the *β*-lactam carbonyl oxygen to facilitate nucleophilic attack and stabilise the negative charge developed on this oxygen in the tetrahedral intermediate anion. The metal-ion also lowers the pKa of the directly co-ordinated water molecule so that the metal-bound hydroxide ion is a better nucleophile than water and is used to attack the *β*-lactam carbonyl carbon. An intrinsic property of binuclear metallo hydrolytic enzymes that depend on a metal-bound water both as the attacking nucleophile and as a ligand for the second metal-ion is that this water molecule, which is consumed during hydrolysis of the substrate, has to be replaced to maintain the catalytic cycle. With MBL this is reflected in some unusual kinetic profiles.

## 1. INTRODUCTION

All *β*-lactam antibiotics, such as penicillins (1) and cephalosporins (2), contain the four-membered *β*-lactam ring which occurs relatively rarely in nature, therefore it is not surprising that the biological activity of these compounds should be attributed to the expected enhanced chemical reactivity of the *β*-lactam ring [1]. It was suggested early that the antibiotic's activity was due to the inherent strain of the four-membered ring or to reduced amide-resonance. The nonplanar butterfly shape of the penicillin molecule (3) was expected to reduce amide-resonance and thus increase the susceptibility of the carbonyl group to nucleophilic attack, compared with normal, planar amides [1]. However, it was shown later that there is little evidence to confirm that the reactivity of *β*-lactams in penicillins and cephalosporins is due to an unusually strained or an amide-resonance inhibited *β*-lactam [2]. In fact, the rate of alkaline hydrolysis of the simple *β*-lactam (4) is only 3-fold greater than that of (5).


(1)
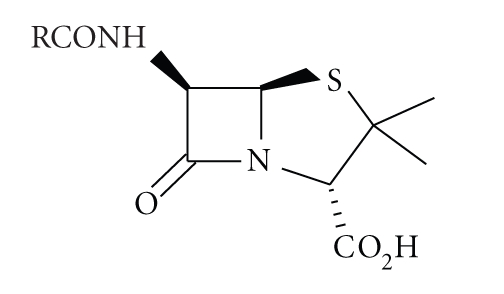

(2)
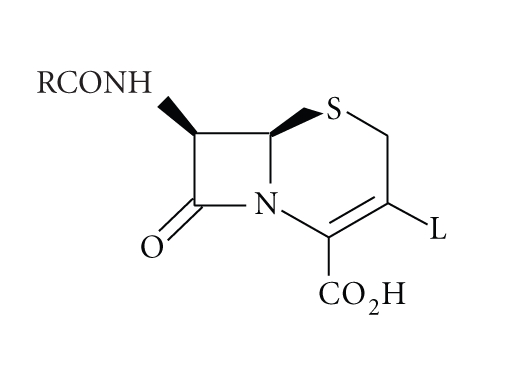

(3)
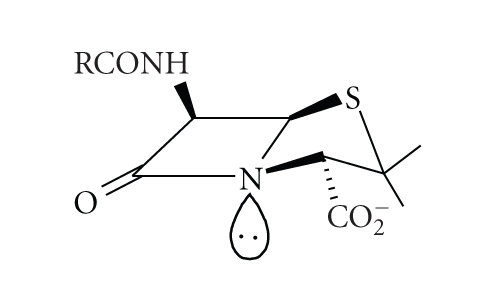

(4)
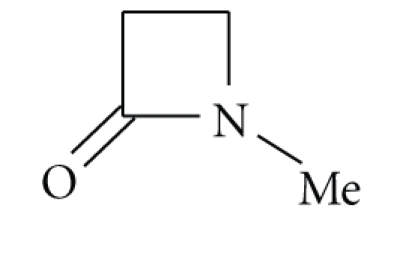

(5)
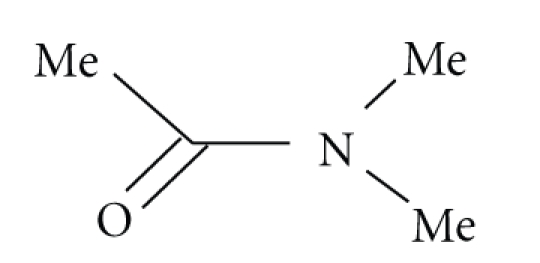



**Scheme 1:**
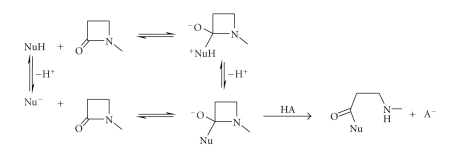


Most nucleophilic substitution reactions of *β*-lactams involve initial covalent bond formation between the carbonyl carbon the of *β*-lactam and the attacking nucleophile followed by C–N bond fission of the *β*-lactam (Scheme 1).

This type of reaction is a two-step process and involves the reversible formation of a tetrahedral intermediate [1, 2]. Contrary to expectations, opening the four-membered ring is not a facile process [3] and, indeed, in many of these nucleophilic substitution reactions, the rate limiting step is not the first addition step but a subsequent one which may sometimes even be ring opening itself [1, 2, 4–8].

Another interesting difference between nucleophilic substitution in penicillins and peptides/amides is the preferred direction of attack and the geometry of the initially formed tetrahedral intermediate. It is usually assumed that nucleophilic attack on the carbonyl carbon of a planar peptide will generate a tetrahedral intermediate with the lone pair on nitrogen *anti* to the incoming nucleophile, (6), (7). Conversely, nucleophilic attack on the *β*-lactams of penicillins occurs from the least hindered *α*-face (*exo*) so that the *β*-lactam nitrogen lone pair is *syn* to the incoming nucleophile in the tetrahedral intermediate (8) [9]. This has obvious consequences for the placement of catalytic groups—particularly, if catalysis involves coordination to metal-ions [10].


(6)
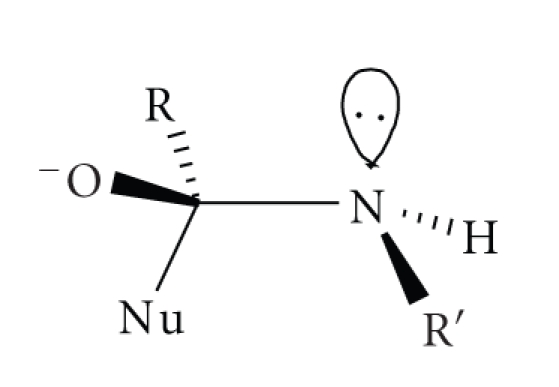

(7)
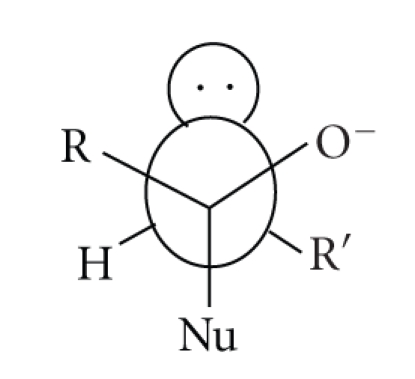

(8)
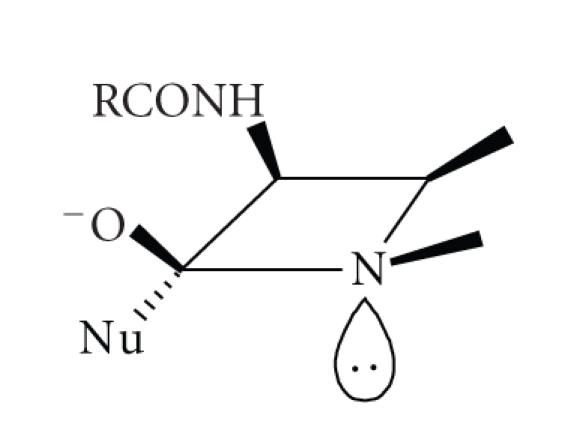



## 2. *β*-LACTAMASES

It is more than sixty years since *β*-lactams were first introduced into clinical practice, but their therapeutic importance remains extremely high and they account for around half of total antibiotic prescriptions [11]. Given the rapid replication rates of bacteria against this selective pressure, it is not surprising that mechanisms of resistance continue to evolve and spread amongst pathogenic bacteria. Although there are several mechanisms of *β*-lactam resistance including modification of the target (transpeptidases), reduction in permeability, and efflux of the antibiotic, the major reason for resistance is antibiotic inactivation by the action of a variety of *β*-lactamases that are found in a large number of clinically important pathogens [12–14]. This number presently exceeds 500 and is growing (http://www.lahey.org/studies/).

**Scheme 2:**
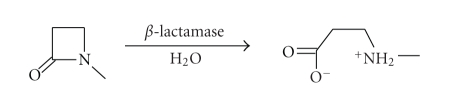


*β*-Lactamases catalyse the hydrolysis of the *β*-lactam of penicillins (1) and cephalosporins (2) to give the ring opened and bacterially inert *β*-amino acid (Scheme 2) [15].

The main mechanistic division of *β*-lactamases is into serine enzymes and zinc enzymes [15]. The former have an active site serine residue and the catalytic mechanism involves the formation of an acyl-enzyme intermediate. The metallo enzymes appear to involve coordination of the substrate and intermediates to the active site metal-ion. On the basis of their amino acid sequences, the serine *β*-lactamases are subdivided into three classes: A, C, and D, whereas the class B *β*-lactamases consist of the zinc enzymes [15].

## 3. METALLO *β*-LACTAMASES

Class B *β*-lactamases or metallo-*β*-lactamases (MBLs) require zinc-ions to catalyse the hydrolysis of *β*-lactams and have no sequence or structural homology to the serine *β*-lactamases. They exhibit a broad spectrum substrate profile catalysing the hydrolysis of a wide range of *β*-lactam antibiotics including penicillins, cephalosporins, carbapenems, cephamycins, and even some mechanism-based inhibitors of class A *β*-lactamases [16]. The first metallo-*β*-lactamase to be discovered was produced by an innocuous strain of *Bacillus cereus*, but in the last 20 years, MBL-mediated resistance has appeared in several pathogenic strains and is being rapidly spread by horizontal transfer, involving both plasmid and integron-borne genetic elements [17]. MBLs represent a huge potential clinical threat to the *β*-lactam antibiotic therapy as presently there are no clinically useful inhibitors for them.

MBLs can be divided in three subclasses: subclass B1, B2, and B3 based on their aminoacid sequences, substrate profile, and metal-ion requirement [18, 19]. Subclass B1 is the largest and contains several well-studied *β*-lactamases: BCII from *Bacillus cereus,* CcrA from *Bacteroides fragilis*, IMP-1, SPM-1 from *Pseudomonas aeruginosa,* and BlaB from *Cryseobacterium meningosepticum*. These enzymes efficiently catalyse the hydrolysis of a wide range of substrates such as penicillins, cephalosporins, and carbapenems. BCII catalyses the hydrolysis of penicillins at significantly higher rates than cephalosporins and carbapenems, but CcrA does not show this preference, although both enzymes exhibit lower K_m_ values for cephalosporins [20].

The most common representatives of subclass B2 are CphA from *Aeromonas hydrophila* and ImiS from *Aeromonas veronii*, which preferentially hydrolyze carbapenems, for example, imipenem and meropenem, but have poor activity against penicillins and cephalosporins [20, 21]. Finally, subclass B3 contains the only known tetrameric zinc *β*-lactamase, the L1 enzyme from *Stenotrophomonas maltophilia,* and the monomeric FEZ-1 from *Legionella gormanii*. Both enzymes hydrolyze a wide range of *β*-lactam antibiotics [22, 23] with L1 having higher catalytic rate constants (k_cat_) for penicillins compared with FEZ-1, which shows higher k_cat_ values for cephalosporins. Generally, the K_m_ values are smaller for L1 than for FEZ-1. However, mutation of a methionine residue in L1, which is important for the subunit interaction, gives a monomeric enzyme with significantly higher K_m_ values and, except for nitrocefin, smaller k_cat_ values compared with the wild-type tetramer [24].

### 3.1. The number of zinc-ions

A major problem with understanding the mechanism of MBLs is the number of zinc-ions required for catalysis, which has been addressed by studying MBLs under both equilibrium and kinetic conditions. The crystal structures of several MBLs have been determined by X-ray diffraction and they all show a similar *α* *β*/*β* *α* sandwich fold, which was first seen with MBLs [25], but since recognised in other enzymes, such as glyoxalase II, aryl sulfatase, and cAMP phosphodiesterase, which have now become members of the MBL fold superfamily [26]. The active site of MBLs is situated at the bottom of a wide shallow groove between two *β*-sheets and has *two* potential zinc-ion binding sites at the active site, often referred to as sites 1 and 2 [27–29]. The zinc ligands in the two sites are not the same and are not fully conserved between the different MBLs. Table 1 shows the enzyme residues involved in zinc coordination in the two binding sites in the subclasses B1, B2, and B3 [26].

In the subclass B1, such as the *Bacillus cereus* enzyme BCII, the zinc in site 1 (the histidine site or His_3_ site) is tetracoordinated by the imidazole groups of three histidine residues (116, 118, and 196) and a water molecule, Wat_1_. In site 2 (or the Cys site) the metal is pentacoordinated by His263, Asp120, Cys221, and one water molecule; the fifth ligand at site 2 is carbonate or water, often referred to as the apical water, or Wat_2_ [27, 28, 30], although this is missing in structures with inhibitors bound [31]. The two metal-ions are relatively close to each other, but the distance between them varies from 3.4 to 4.4 Å in different structures of the BCII and CcrA enzymes. Several structures of the CcrA enzyme show a bridging water ligand between the two metals, which is thought to exist as a hydroxide-ion [32]. In a structure of BCII determined at pH 7.5 that contains two zinc-ions there is also a similar bridging water molecule, but in structures of this enzyme at lower pH this solvent molecule is strongly associated to the zinc in site 1 [30].

The two conserved zinc binding sites in MBLs have different metal-ion affinities. For example, the BCII enzyme from *Bacillus cereus* has very different dissociation constants for the two metal binding sites. The first crystal structure, obtained at low pH [25], indicated only one zinc-ion bound to the histidine site, but equilibrium dialysis studies showed two binding events, with a dissociation constant of 0.3 *μ*M for the first zinc-ion (K_mono_) and 3 *μ*M for the second zinc-ion (K_bi_) [33]. Later metal binding studies by fluorescence spectroscopy using a chromophoric chelator reported a K_mono_ of 0.62 nM and a K_bi_ of 1.5 *μ*M [34]. As the conditions of the experiments were similar for the two studies, the large discrepancy in K_mono_ may be due to the fact that different strains of BCII enzyme from *Bacillus cereus* were used. The dissociation constants K_mono_ and K_bi_ have been determined from the steady state rates of hydrolysis of imipenem in the presence of EDTA as a metal-ion buffer at different zinc concentrations and K_mono_ was found to decrease significantly, from nM to pM, in presence of substrate, whereas K_bi_ decreased only by two fold [35]. This led to the suggestion that the monozinc enzyme is responsible for the catalytic activity under physiological conditions, where the concentration of the “free” Zn^2+^ is in the pM or even fM region. For most substrates, the reported catalytic activity of the monozinc BCII was about two fold lower than that of the binuclear enzyme. Conversely, the other class B1 enzyme, CcrA from *Bacteroides fragilis,* binds both zinc-ions very tightly [36]. Despite the very close similarity with BCII, CcrA has much higher affinity for the second zinc-ion, probably due to the fact that in CcrA, a cysteine residue replaces the positively charged arginine 121 found in BCII. However, replacing Arg 121 in BCII by a Cys shows no increase in the affinity for the second zinc-ion [37].

Early kinetic studies of CcrA led to the proposal that *both* the mono and the dinuclear forms of the enzyme were catalytically active, with slightly different activities, at physiological pH [38]. However, later studies showed that only the dinuclear species was active and that the previously observed “monozinc” CcrA was a mixture of the dizinc and the apo (metal free) enzyme [39]. Class B2 metallo *β*-lactamases are catalytically active with one bound zinc-ion and the binding of the second zinc-ion inhibits the enzyme noncompetitively, with a K_i_ of 50 *μ*M [40]. The dissociation constant of the first zinc-ion was found to be 7.0 and 1.2 pM in the absence and presence of substrate (imipenem), respectively [35]. Subclass B3 MBLs, L1, and FEZ-1 bind both metal-ions tightly, the dissociation constants are in the nM region, and are fully catalytically active in binuclear form [23, 41].

### 3.2. The catalytic role of zinc

Zinc is an essential trace element and the second most abundant transition metal found in living sytems. Its role in catalysis is related to its ability to participate in tight, but readily exchangeable ligand binding and its exceptional flexibility of its coordination number and geometry [42]. In addition, zinc shows no redox properties and this facilitates its evolution in living systems without the risk of oxidative damage. Finally, its intermediate hard-soft behaviour allows it to bind a variety of atoms, as seen, for example, in the second binding site of class B1 MBLs which involve nitrogen, oxygen, and sulphur as ligands. The Lewis acidity, flexible geometry, and coordination number and the lack of redox properties make zinc an ideal metal cofactor for many enzymes. The small energy difference between 4, 5 or 6 coordination geometries and the rapid exchange of the kinetically labile zinc-bound water molecule are important features in all zinc hydrolases including MBLs.

**Scheme 3:**
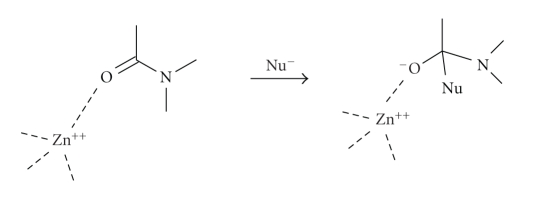


**Scheme 4:**
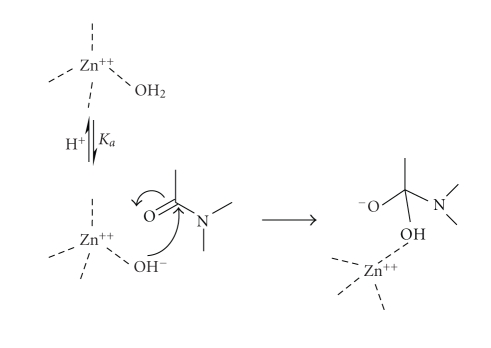


**Scheme 5:**
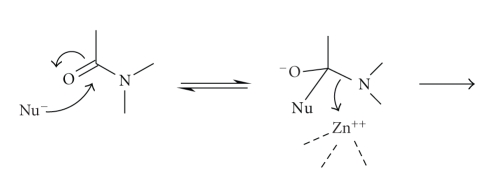


**Scheme 6:**
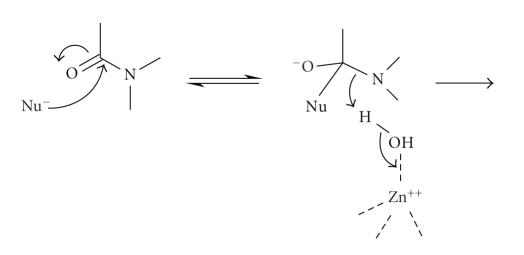


MBLs are a subclass of metallo-proteases and many mechanistic considerations are applicable to both groups. There are many potential mechanistic roles for the metal-ion in metallo-proteases and MBLs, although they may well vary from enzyme to enzyme [42, 43]. It is commonly suggested that the metal-ion acts as a Lewis acid by coordination to the peptide carbonyl oxygen giving a more electron deficient carbonyl carbon which facilitates nucleophilic attack. The metal-ion thus stabilises the negative charge developed on the carbonyl oxygen of the tetrahedral intermediate anion (Scheme 3). Many metallo-proteases have a water molecule directly coordinated to the metal-ion which may act as the nucleophile to attack the carbonyl carbon. Here, the role of the metal-ion is to lower the pK_a_ of the coordinated water so that the concentration of metal-bound hydroxide ion is increased relative to bulk solvent hydroxide-ion at neutral pH and, furthermore, although this coordinated hydroxide-ion is different in nature than the simple solvated ion, it is a better nucleophile than water (Scheme 4). These two steps shown in Schemes 3 and 4 relate to formation of the tetrahedral intermediate and although little attention is normally given to the mechanism of the breakdown of the tetrahedral intermediate, C–N bond fission is the most energetically difficult process in peptide hydrolysis. This could be facilitated by direct coordination of the departing amine nitrogen to the metal-ion (Scheme 5) which is, in fact, the mechanism adopted for the simple zinc-ion-catalysed hydrolysis of penicillin [10]. Alternatively, a metal-bound water could act as a general acid catalyst protonating the amine nitrogen leaving group to facilitate C–N bond fission (Scheme 6). Despite intense mechanistic studies, the detailed roles of the metal-ion in metallo-proteases and MBLs remain controversial and distinguishing between the relative importance of the possible roles for zinc is complex.

The effective positive charge on the zinc-ion depends on the number and nature of its ligands and for those with an ionisable hydrogen, zinc lowers their pK_a_ and the ionised ligand obviously is better at neutralising the positive charge density on the metal. The pK_a_ of water is 15.7, but when bound to zinc in aqueous solution it is lowered to 9.5, but is there evolutionary pressure to lower the pK_a_ of the zinc-bound water in an enzyme even further? This could be achieved by replacing say a negatively charged carboxylate ligand by a neutral histidine. For example, in the opposite direction, changing the histidine bound to zinc in carbonic anhydrase to aspartate increases the pK_a_ of the zinc-bound water from 6.8 to ≥ 9.6 [45]. Whether it is better to have a higher or lower pK_a_ metal-bound water for a faster reaction and more efficient catalysis depends on the role of the zinc-ion. A low pK_a_ implies a more electron deficient metal-ion centre which would give a better Lewis acid to stabilise the negative charge developed on the oxyanion of the tetrahedral intermediate. Conversely, a high pK_a_ for the metal-bound water implies a weaker Lewis acid and so the zinc-ion will be less efficient at stabilising the tetrahedral intermediate. A low pK_a_ for the metal-bound water implies that the hydroxide-ion resulting from ionisation is more “tightly bound” and stabilised which, although it becomes the dominant species even at low pH, corresponds to a more weakly nucleophilic hydroxide-ion. For example, if the pK_a_ of the zinc-bound water is about 5 then the nucleophilicity of the metal bound hydroxide-ion is only similar to that of a carboxylate anion. If a major role of the metal-ion is to provide a better nucleophile than water then the net effect depends on the relative importance of concentration and the dependence of the rate upon nucleophilicity. If the pK_a_ is “too high”, metal-coordinated water will be the dominant species over the desired pH range but deprotonation will give a more nucleophilic metal-bound hydroxide. How reactivity changes with changing pK_a_ and pH will depend on the susceptibility of the rate of reaction to the basicity of the nucleophile—the hydroxide-ion bound to the metal—as indicated by the Bronsted *β*_nuc_ value.

Sometimes it is suggested that part of the enzyme mechanism involves general base catalysis to remove the proton from the zinc-bound water but this only becomes necessary if the pK_a_ is little changed from that of bulk water. A problem with the commonly accepted mechanism for metallo-proteases of rate-limiting deprotonation of zinc-bound water concerted with nucleophilic attack is the pK_a_ of this water. Coordination to zinc(II) generally lowers the pK_a_ of water to 5–9 depending on the number and type of other ligands. The concentration of zinc-bound hydroxide ion is therefore quite high over the normal pH range studied. There is little or no *catalytic* advantage in having a general base to remove a proton in this pre-equilibrium step! Even if the pK_a_ of the zinc(II)-bound water is about 9 then 10% of the species already exists in the fully deprotonated form at pH 8 and 1% at pH 7. Presumably, the deprotonated form is a much better nucleophile than the species which is only partially deprotonated and there would be no catalytic advantage of the general base-catalysed mechanism.

**Scheme 7:**
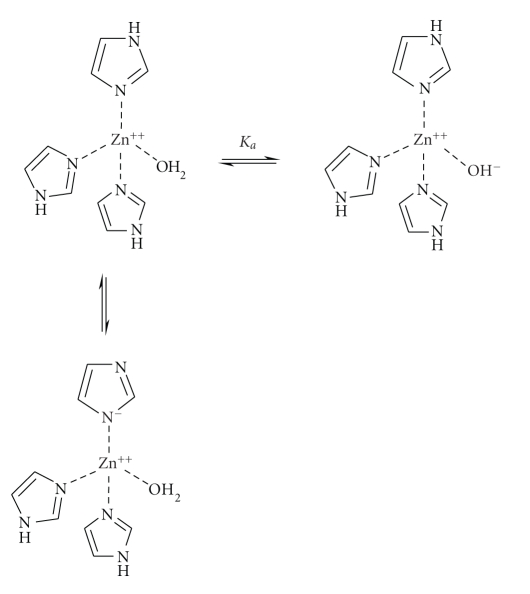


In aqueous solution, zinc is coordinated to six water molecules and the pK_a_ of the zinc-bound water is 9.5. However, within a protein this pK_a_ can be changed significantly because of the environment and directly bonded ligands to the metal—for example, the zinc of *B. cereus* *β*-lactamase is coordinated to three protein ligands in the zinc1 site—His 116, His 118, and His 196 and a water molecule [30]. The reduced coordination number of four in the zinc1 site of *β*-lactamase reduces the pK_a_ to less than 6. In principle, one or more of the imidazole residues could be ionised, the pK_a_ for proton loss from imidazole is similar to that of water (about 14). However, NMR evidence suggests that the imidazole of the bound histidines are neutral [46, 47] and the most likely group to ionise is, therefore, the bound water (Scheme 7).

One way to investigate the role of the zinc in catalysis is to modify the pK_a_ of the zinc-bound water by changing the ligands or the metal-ion. If the activity of the resulting enzyme *increases* as the pK_a_ of the zinc-bound water ligand *decreases,* this would suggest that a high positive charge density on the metal-ion facilitates catalysis and that the zinc coordination to the carbonyl oxygen and stabilisation of the negative charge developed on this oxygen following nucleophilic attack is important. A lower pK_a_ of zinc-bound water indicates a more electrophilic zinc which is better at stabilising negative charge giving rise to a better catalyst. Stabilisation of the intermediate anion must be more important than the nucleophilicity of the zinc hydroxide. Conversely, if activity *increases* with *increasing* pK_a_ of the zinc-bound water, that is, increasing basicity of the of the zinc-bound hydroxide ion, then this would indicate a greater role for the metal-ion in controlling the nucleophilicity of the hydroxide-ion compared with its role in stabilising the negative charge development on the carbonyl oxygen.

**Scheme 8:**
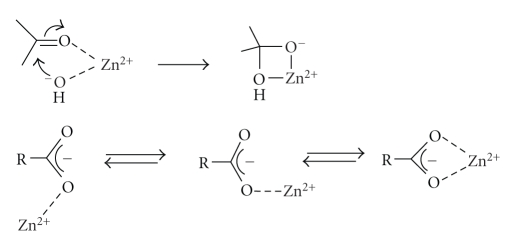


The *dual* role of zinc in metallo-proteases, that is, acting as a Lewis acid in polarising the carbonyl bond and as source of nucleophilic hydroxide, requires formally the formation of an apparently strained four-membered ring (Scheme 8). However, this coordination occurs in the bidentate complexes of zinc with carboxylate anions. Monodentate complexes between zinc and a carboxylate anion exist predominantly as the *syn* stereoisomer due to the more basic *syn* lone pair and favourable opposition of the C=O and O–Zn dipoles. The coordination adopted for the *syn* conformation is similar to a bidentate one which is favoured by electrostatic interactions [48].

**Scheme 9:**
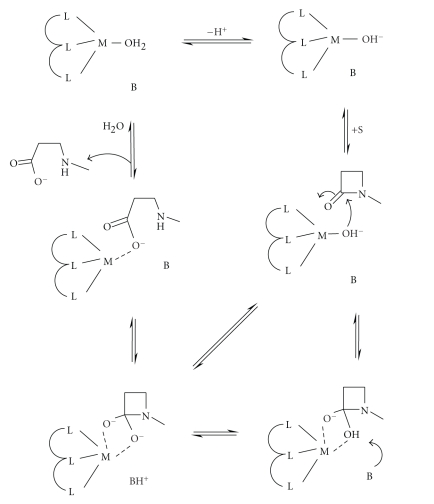


One final point to consider for the hydrolysis of *β*-lactams by metallo-*β*-lactamases is that if the zinc bound hydroxide-ion is the nucleophile then it is *consumed* during turnover. Consequently, regeneration of the catalyst requires the coordination of a new water molecule to the active site zinc. For example, if product release occurs through displacement by water or is introduced in a separate step following product release, *deprotonation* must occur to generate a catalytically active species (Scheme 9). It is conceivable that one of these steps may become kinetically significant under some conditions, particularly if the water is used both as a nucleophile and as a bridging ligand in binuclear enzymes.

### 3.3. Mechanisms

**Scheme 10:**
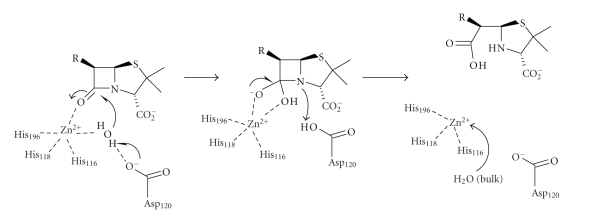


Although the metal-ion requirement in MBLs catalysed hydrolysis of *β*-lactam antibiotics is still a matter of debate, catalytic mechanisms have been proposed for both the mono- and binuclear enzymes. The first catalytic mechanism suggested for BCII [25] (Scheme 10) was based on its crystal structure at a resolution of 2.5 $\overset{\prime }{Å}$, where only one zinc-ion was bound to the enzyme, in the histidine site. The main features of this mechanism are similar to those often proposed for zinc peptidases such as carboxypeptidase A and thermolysin. Following substrate binding, the zinc- bound water molecule, deprotonated by the Asp120 residue, attacks the carbonyl centre with the formation of a negatively charged tetrahedral intermediate, which is stabilised by its interactions with the metal-ion. The Asp120 residue donates a proton to the nitrogen and C–N bond cleavage occurs, followed by product dissociation from the enzyme active site. The main disadvantage of this model is the unnecessary step of removing a proton from the zinc-bound water as it is already ionised, although this may be required during turnover.

**Scheme 11:**
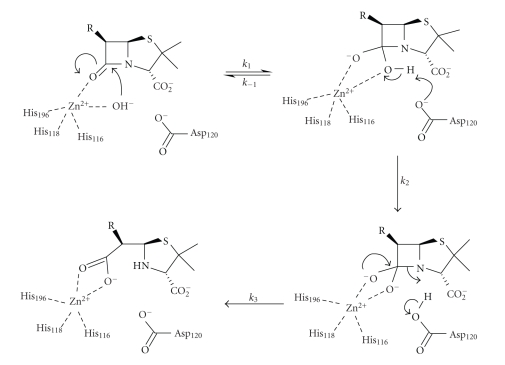


The pH-rate profile for the BCII catalysed hydrolysis of benzylpenicillin and cephaloridine [49] was taken to indicate that the zinc-ion-bound water has a low pK_a_ of <5 and is therefore fully ionised at neutral pH. Nucleophilic attack by the metal-bound hydroxide ion on the carbonyl followed by a proton abstraction from the Asp120 gives a dianionic tetrahedral intermediate (Scheme 11). It was suggested that the same aspartate residue functions as proton donor to facilitate C–N bond fission, and either step k_2_ or k_3_ could be rate limiting. A dianionic intermediate assists *β*-lactam ring opening and generates a carboxylate anion rather than the undissociated acid.

**Scheme 12:**
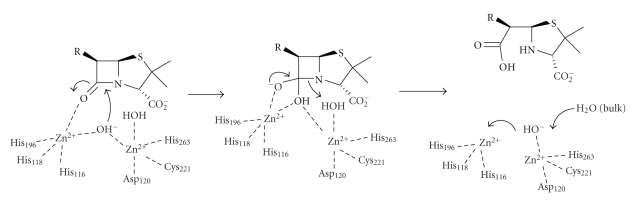


Based on the crystal structure of CcrA MBL from *Bacteroides fragilis* and on models of *β*-lactam substrates bound in the enzyme active site, a mechanism of hydrolysis for the dizinc enzyme has been suggested to be analogous to mechanisms proposed for other binuclear metallo-hydrolases [50]. In this mechanism (Scheme 12), the bridging hydroxide-ion is responsible for the nucleophilic attack which results in a negatively charged intermediate stabilised by the oxyanion hole of the enzyme. The apical water molecule bound to zinc is optimally positioned to donate a proton to the leaving nitrogen, and the newly formed hydroxide-ion moves to occupy the vacated Wat1 site, followed by product dissociation from the enzyme active site. The original proposal involved zinc coordination to the *β*-lactam nitrogen, but this is unlikely because of its relatively low electron density due to amide-resonance. However, nitrogen binding to zinc is more likely once the tetrahedral intermediate is formed because of its increased basicity [10].

These studies used the unusual *β*-lactam nitrocefin as substrate for CcrA, which, because of the chromophoric product, allowed rapid scanning and single-wavelength stopped-flow studies to reveal the accumulation, during turnover, of an enzyme bound intermediate with an intense absorbance at 665 nm [51, 52]. This was postulated to be an enzyme intermediate in which the leaving nitrogen atom is not protonated during the cleavage of the C–N bond and remains negatively charged (9). The anion is stabilised by extensive conjugation with the dinitrostyryl substituent in the C_3_ position and by the zinc-ion in the second active site and so is an atypical leaving group. Most amine anions are very unstable and are unlikely to be expelled without N-protonation.


(9)
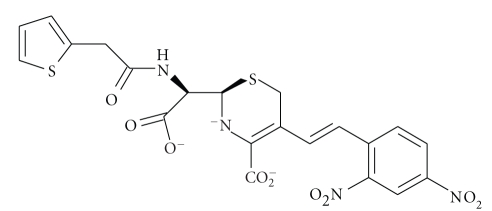



A similar intermediate has been observed during the class B3 L1 enzyme-catalysed hydrolysis of nitrocefin, and, as in the case of CcrA, its breakdown was rate-limiting [53]. However, stop-flow studies of the tryptophan fluorescence revealed that the mechanism of nitrocefin hydrolysis by binuclear metallo-*β*-lactamases may be unusual and cleavage of the *β*-lactam amide bond is the rate determining step for the breakdown of the majority of *β*-lactam substrates by the L1 enzyme [54].

In contrast to these results, no accumulation of an anionic intermediate could be detected in reactions catalysed by BCII and the rate of substrate depletion and product formation are similar [55]. However, for the BCII-catalysed hydrolysis under cryoenzymological conditions, a slightly red-shifted intermediate (440 nm) was observed, which was suggested to be a nitrocefin-like tetrahedral intermediate [56].

**Scheme 13:**
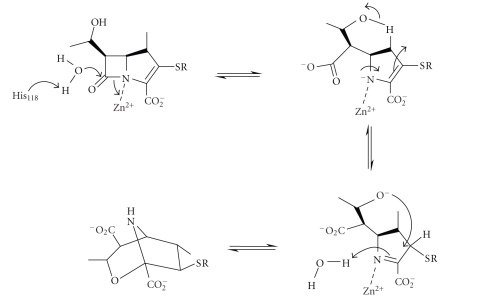


An unusual mechanism has been proposed for subclass B2 metallo-*β*-lactamase CphA from *Aeromonas hydrophila* based on the crystal structures of the free enzyme and of a reaction intermediate between the enzyme and the substrate meropenem (Scheme 13) [57]. Nucleophilic attack is performed by a water molecule not coordinated to zinc, but activated by general base catalysis by His118, followed by C–N bond fission which occurs prior to nitrogen protonation. This proposal uses zinc as a Lewis acid to facilitate C–N bond fission and to stabilise the leaving group—a mechanism proposed much earlier based on model studies [10]—whereas most mechanisms use the zinc-ion to act as a Lewis acid to stabilize oxyanion formation from the *β*-lactam carbonyl oxygen. Rearrangement of the proposed intermediate amine anion, whose negatively charged nitrogen is stabilised by an interaction with the zinc-ion, gives the bicyclic intermediate shown (Scheme 13), which is observed in the crystal structure, although this rearrangement may not occur in the active site of the enzyme. A solvent molecule replacing the water used in the nucleophilic attack protonates the nitrogen and releases the final product.

### 3.4. Mutation studies with MBLs

Many mutant enzymes from the three subclasses of MBLs have been produced and characterised. In general, the residues substituted have been the metal ligands, those providing the framework for orienting the metal ligands or those thought to interact with the substrates. For BCII, replacing the histidine residues in the first binding site with serine resulted in decrease of catalytic activity, although the affinity for zinc was not significantly altered [58]. The decrease in activity appeared as due to an increase in K_m_ which was rationalised on the basis that, in absence of a His ligand, there is a higher degree of flexibility of the substrate inside the catalytic site.

The replacement of the Cys221 residue with alanine or serine in the active sites of BCII [58], CcrA [59], and IMP-1 [60] gives a drastic decrease in the rate of hydrolysis at low zinc concentrations, but the mutants are “reactivated” by the addition of excess zinc, at least in case of BCII and IMP1. It was proposed that the presence of Cys has a crucial importance for the catalytic activity of the monozinc enzyme but not for the dinuclear species [58].

Substituting the zinc 2 ligand Asp120 in BCII with Asn reduces the catalytic activity by more than 100 fold, but does not impair the binding of the second zinc-ion [58]. Although these results support the hypothesis that Asp120 plays an important role in catalysis, the significant catalytic activity of the mutants is inconsistent with Asp playing the role of a general base. The same amino acid substitution in subclass B3 MBL enzyme L1 led to an average 10 fold decrease in catalytic activity in the binuclear mutant [61]. It is interesting that for the hydrolysis of nitrocefin, this mutant shows an unusually large kinetic solvent isotope effect (KSIE) of 5.36 compared with the wild type (KSIE = 2.08) and other aspartate mutants: Asp120Cys and Asp120Ser (KSIE < 2). It is concluded that the Asp120 residue is essential for the catalytic activity in the orientation of the bridging group of the L1 enzyme, in the protonation step of breakdown of the tetrahedral intermediate, rather than in its formation involving nucleophilic attack, as previously suggested [62].

The mutation of the second shell ligand, Arg121Cys, in BCII gives an enzyme with a lower affinity for the second metal-ion compared to the wild type [58]. Moreover, the electronic spectrum of the dicobalt Arg121Cys BCII mutant is identical to that of the wild-type enzyme indicating that the mutation has not altered the metal coordination sphere. The catalytic activity of the mutant is only two fold lower than that of the wild type for both the mono- and dinuclear forms of the enzyme. The Cys104Arg mutation in CcrA led to a binuclear enzyme with significantly decreased activity and which, unlike the wild type, can have one bound metal-ion removed by a chelator [39]. Presteady state kinetics suggested a change in the rate limiting step in the hydrolysis of nitrocefin, from nitrogen protonation in the wild type, to C–N bond fission in the mutant, which showed a catalytic rate constant similar to that of the dizinc BCII enzyme. The monozinc form of the mutant showed a k_cat_ value similar to that of the monozinc BCII, for nitrocefin hydrolysis, with a further decrease of the rate of C–N bond fission compared with the dizinc mutant, but with a similar protonation rate. Based on these observations it was concluded that both the replacement of the arginine residue and the introduction of the second metal-ion are evolutionary tools for accelerating C–N bond fission in MBL catalysed hydrolysis of *β*-lactams. However, an important role in lowering the energy barrier for breaking the C–N bond must come from other enzyme structural rearrangements, since the reciprocal, that is, the sole replacement of the arginine by a cysteine residue and the insertion of a second zinc-ion in BCII, only marginally (two fold) improves the catalytic activity [55].

Another interesting mutagenesis study has been carried out on class B2 CphA enzyme from *Aeromonas hydrophila*, which is a special case of an MBL, both regarding its narrow substrate profile and its metal-ion requirement [63]. The Asn116 residue in site one was mutated to a histidine in an attempt to create a B1 type first binding site; this mutation changed the properties of the CphA enzyme towards a broader substrate profile (characteristic to subclass B1), as both penicillins and cephalosporins became significantly better substrates. Moreover, the activity of the Asn116His mutant increased with increasing metal-ion concentration for the hydrolysis of benzylpenicillin and cephaloridine, as opposed to imipenem, where addition of zinc to both the mutant and the wild type lead to noncompetitive inhibition. The Cys221Ser and Cys221Ala mutants were seriously impaired in their ability to bind the first zinc-ion and were nearly completely inactive indicating a major role for Cys221 in binding the catalytic metal-ion.

### 3.5. Metal ion substitution

The exchange of the spectroscopically silent zinc in zinc enzymes with probes, such as cobalt, copper, and cadmium, enables the study of the metal interactions in the enzyme active site with its ligands, substrates, and inhibitors of the metallo-enzyme using techniques such as electronic spectroscopy, NMR, EPR, and perturbed angular correlation (PAC) spectroscopy. The zinc of metallo *β*-lactamases can be exchanged with cadmium, cobalt, and manganese to give catalytically active enzymes. The use of a combination of NMR and PAC spectroscopy to study cadmium binding to *B. cereus* MBL has revealed a rapid intramolecular exchange of the metal between the two sites in the monocadmium enzyme and negative cooperativity in metal binding [64]. The enzyme inhibitor (R)-thiomandelate induces a very strong positive cooperativity for binding the second cadmium cation [65].

The metal-ion environment of cobalt-substituted metallo *β*-lactamases has been studied by UV-vis spectroscopy, NMR, and ESR. The UV-vis spectra of cobalt-substituted class B1 metallo-*β*-lactamases CcrA from *Bacteroidis fragilis* [36, 66] and BCII from *Bacillus cereus* [67, 68] show similar features with an intense S to Co(II) ligand to metal charge transfer transition (LMCT) band at 340 and 348 nm and four characteristic d-d transition bands between 500 and 650 nm. The spectrum of Co-BCII in the visible region is also very similar to that of Co(II) carbonic anhydrase at alkaline pH [69], suggesting that the contributions from the His_3_ metal centre dominate this part of the spectrum of Co substituted class B1 MBLs.

On titration of monozinc BCII with Co(II), the spectrum shows an LMCT band, but without the four ligand field bands which suggests that zinc binds to the His_3_ site, while cobalt preferentially occupies the Cys site in the hetero-substituted CoZn-enzyme [67]. A similar discrimination between metals in occupying the two binding sites in BCII was noted for the CdZn-enzyme, where zinc also binds in the His_3_ site and Cd to the Cys site [64]. Studies of cobalt binding to apo BCII, using HEPES as a buffer and no added sodium chloride, showed that increasing the ratio Co(II)/enzyme above one resulted in a decrease of the charge transfer band at 344 nm and the appearance of an additional charge transfer band at 383 nm. This has been explained by the binding of a second cobalt ion to the monocobalt BCII species and the existence of two different Co(II) LMCT band positions, one for the mononuclear (344 nm) and one for the binuclear enzyme (383 nm); this latter band disappears upon addition of sodium chloride, accompanied by an increase in absorbance at 344 nm, which led to the suggestion that chloride hinders the binding of a second cobalt ion [34]. More recent data supported by NMR and EPRevidence have shown that the band at 383 nm corresponds to a third, more weakly bound cobalt ion that perturbs the 344 nm signal (the Cys site), without affecting the spectral features of the histidine site [70].

Low-temperature EPR spectra of metallo-*β*-lactamases indicate the presence of high spin, but not coupled, Co(II) ions in a rhombically distorted pentacoordination sphere in Co-substituted BCII [68], a penta/hexacoordination environment in Co-substituted CcrA enzyme [36], a tetracoordination environment for the first cobalt ion, and penta/hexacoordination sphere for the second cobalt ion in Co-substituted ImiS [71]. For this latter enzyme, it has been shown by EPR that the second cobalt ion is magnetically isolated, suggesting a distance of more than 7 Åfrom the first bound cobalt ion. Furthermore, NMR titration of the monocobalt ImiS enzyme with Co(II) indicated that the second cobalt ion is not bound to histidine, as the newly observed resonances are not solvent exchangeable; these findings were taken to indicate that the Zn1 site has no catalytic or metal binding role in ImiS, that Zn2 site binds the metal-ion that is required for catalysis, and a remote, lower-affinity, metal binding site is responsible for the noncompetitive inhibition of the enzyme [72]. From the mechanistic point of view, it has been shown by time dependent UV-vis spectroscopy and fluorescence quenching, using nitrocefin as a substrate, that Co-substituted L1, a class B3 metallo-*β*-lactamase, probably utilises a reaction mechanism similar to that of the native zinc enzyme [54, 73]. Rapid-freeze-quench (RFQ) EPR shows that a short-lived intermediate is a metal-bound species, and the role of the metal-ion in catalysis is similar for nitrocefin, cephalothin, meropenem, and benzylpenicillin [73].

**Figure 1:**
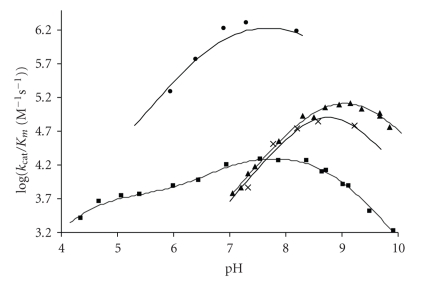
Plot of log k_cat_/K_m_ for (■) ZnBCII-, (•) CoBCII-, (▲) CdBCII-, and (x) MnBCII-catalysed hydrolysis of cephalexin.

The kinetics and mechanism of hydrolysis of the *B. cereus* (BcII) metallo-*β*-lactamase substituted with various metal-ions have been investigated to determine the role of the active site metal-ion [74]. The pH and metal-ion dependence of k_cat_ and k_cat_/K_m_ for the cobalt-substituted BcII catalysed hydrolysis of cefoxitin, cephaloridine, and cephalexin indicate that an enzyme residue of apparent pK_a_ 6.3 is required in its deprotonated form for metal-ion binding and catalysis. The k_cat_/K_m_ for cefoxitin and cephalexin hydrolysis with cadmium-substituted BcII is dependent on two ionising groups on the enzyme: one of pK_a1_ 8.7, required in its deprotonated form, and the other of pK_a2_ 9.3, required in its protonated form for activity. The identity of these residues was determined from the pH-dependence of the competitive inhibition constant, K_i_, of the Cd BcII by L-captopril which showed that the pK_a1_ of 8.7 corresponds to the cadmium-bound water. For the manganese-substituted BcII, the pH-dependence of k_cat_/K_m_ for the hydrolysis of *β*-lactam antibiotics similarly indicated the importance of two catalytic groups: one of pK_a1_ 8.5 which needs to be deprotonated and the other of pK_a2_ 9.4 which needs to be protonated for catalysis; the pK_a1_ was assigned to the manganese-bound water [74]. Interestingly, the metal-substituted enzymes have similar or higher catalytic activities compared with the native zinc enzyme, albeit at pHs above 7 and, for the Co enzyme, at all pHs (Figure 1). With cefoxitin, a very poor substrate for Zn BcII, both k_cat_ and k_cat_/K_m_ increase with increasing pK_a_ of the metal-bound water, in the order Zn<Co<Mn<Cd. A higher pK_a_ for the metal-bound water for cadmium and manganese BCII leads to more reactive enzymes than the native zinc BcII, suggesting that the role of the metal-ion is predominantly to provide the nucleophilic hydroxide, rather than to act as a Lewis acid to polarise the carbonyl group and stabilise the oxyanion tetrahedral intermediate [74].

Given the relatively weak binding of the second zinc in the *B. cereus* (BcII) MBL and the fact that an important ligand holding the metal-ion to the protein is the bridging water which is *consumed* during the catalytic cycle of hydrolysis, it is possible that the second metal-ion could be lost during turnover. In fact, the kinetics of the hydrolysis of benzylpenicillin catalysed by the cobalt substituted *β*-lactamase from *B. cereus* (BcII) are biphasic with an initial burst of product formation followed by a steady-state rate of hydolysis [75]. This was interpreted as being due to a branched kinetic pathway with two enzyme intermediate species, ES^1^ and ES^2^, which have different metal: enzyme stoichiometries. ES^1^ is a dicobalt enzyme intermediate and is catalytic, but is slowly losing one bound cobalt ion during turnover via the branching route, to give the mononuclear and inactive enzyme intermediate ES^2^. The dependence of enzyme activity on pH and metal-ion concentration indicates that only the dicobalt enzyme is catalytically active. The monocobalt enzyme species, formed during the catalytic cycle, is virtually inactive and requires the association of another cobalt ion for turnover. The dicobalt enzyme intermediate is responsible for the direct catalytic route, which is pH-independent between 5.5 and 9.5. The inactivation pathway of metal-ion dissociation occurs by both an acid catalysed and a pH-independent reaction, which is dependent on the presence of an enzyme residue of pK_a_ 8.9 in its protonated form and shows a large kinetic solvent isotope effect (H_2_O/D_2_O) of 5.2, indicative of a rate limiting proton transfer [75]. The pseudo first-order rate constant to regenerate the dicobalt *β*-lactamase from the monocobalt enzyme intermediate has a first-order dependence on cobalt ion concentration. This unusual behaviour is attributed to an intrinsic property of metallo hydrolytic enzymes that depend on a metal-bound water both as a ligand for the second metal-ion and as the nucleophile which is consumed during hydrolysis of the substrate and so has to be replaced to maintain the catalytic cycle [75, 76].

There remain questions regarding the necessity of the binuclear metal centre itself: (i) are metallo-enzyme species responsible for the physiological activity, the binuclear one or the mononuclear one, or are they interchangeable under certain conditions? and if so, (ii) are these changes relevant to any other functions, apart from catalysis, such as regulation of activity? It does seem intrinsically odd in the binuclear enzyme that the bridging hydroxide-ion is used as a nucleophile because of its presumed weak basicity. Hydroxide-ion has three lone pairs, two of which are taken up by metal-ion coordination and one of which is required for nucleophilic attack and covalent bond formation. If this bridging hydroxide-ion is used as the attacking nucleophile is it activated by general base catalysis so that concerted proton removal generates a dianionic tetrahedral intermediate?

## Figures and Tables

**Table 1:** The zinc ligands in class B *β*-lactamases.

Subclass	Zn1 ligands			Zn2 ligands		
B1	His116	His118	His196	Asp120	Cys221	His263
B2	Asn116	His118	His196	Asp120	Cys221	His263
B3	His/Gln116	His118	His196	Asp120	His121	His263
